# Supplementary figures and images for: Urinary titin is not an early biomarker of skeletal muscle atrophy induced by muscle denervation in mice
Source: PLoS One. 2023 Aug 15;18(8):e0289185. doi: 10.1371/journal.pone.0289185 (PMC10426992; doi:10.1371/journal.pone.0289185)

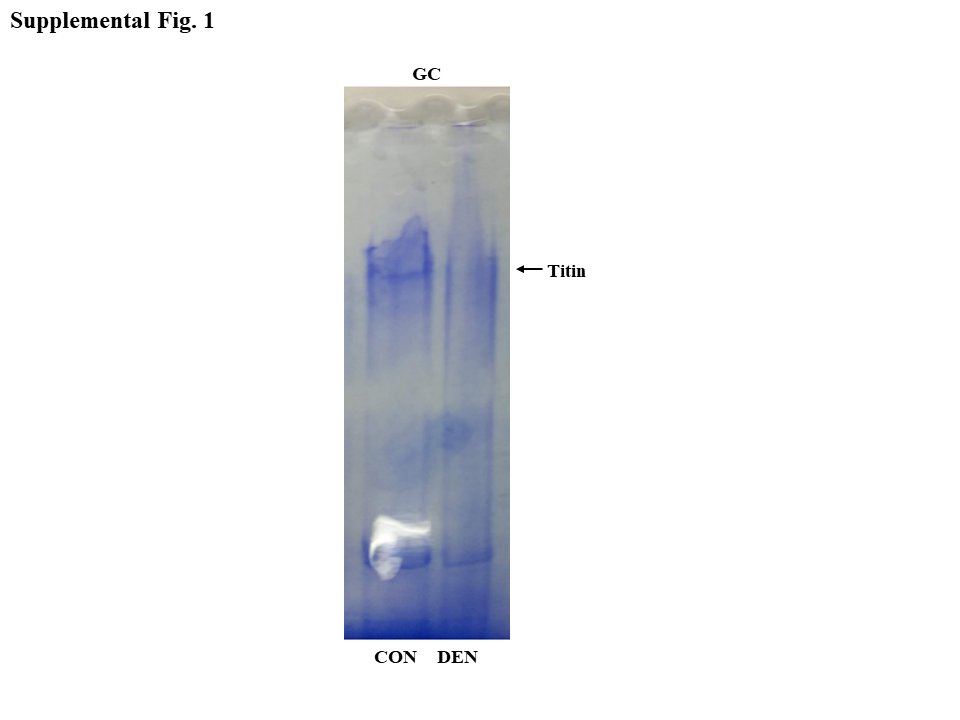

Supplement: S1 Fig — (TIF) [file pone.0289185.s001.tif]

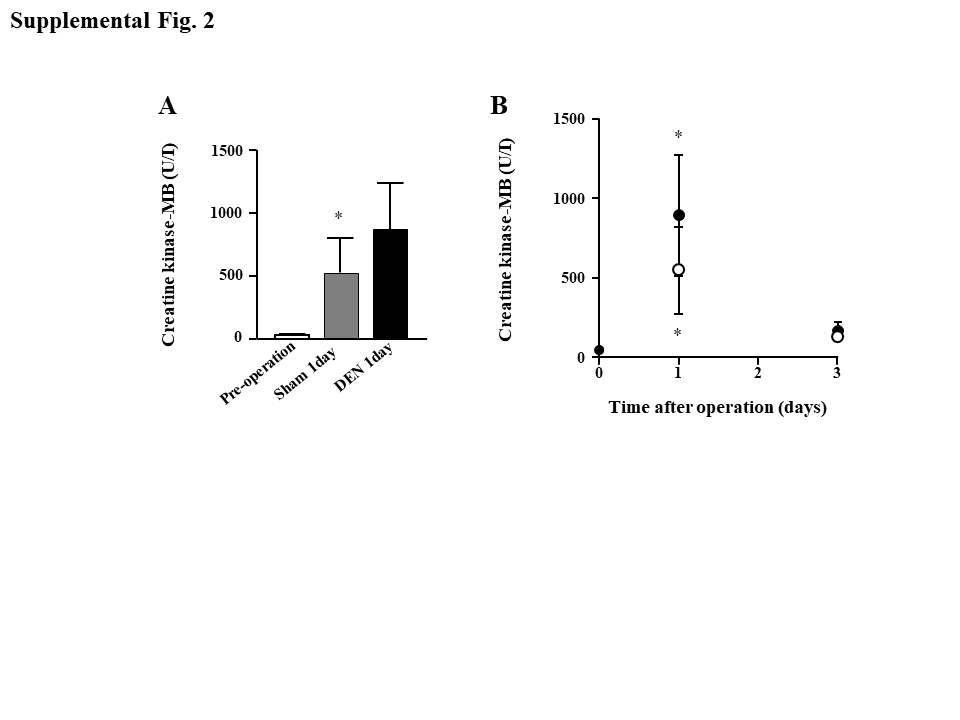

Supplement: S2 Fig — (A) Serum CK-MB levels (U/L) in pre-operation and 1day after sham-operation and denervation. (B) Time course changes in serum CK-MB. Open circles: CON group. Closed circles: DEN group. Data are presented as the means ± standard error of the mean (SEM). n = 4/group. *p < 0.05 by an unpaired t-test (Compared to pre-operation (0 day)). (TIF) [file pone.0289185.s002.tif]

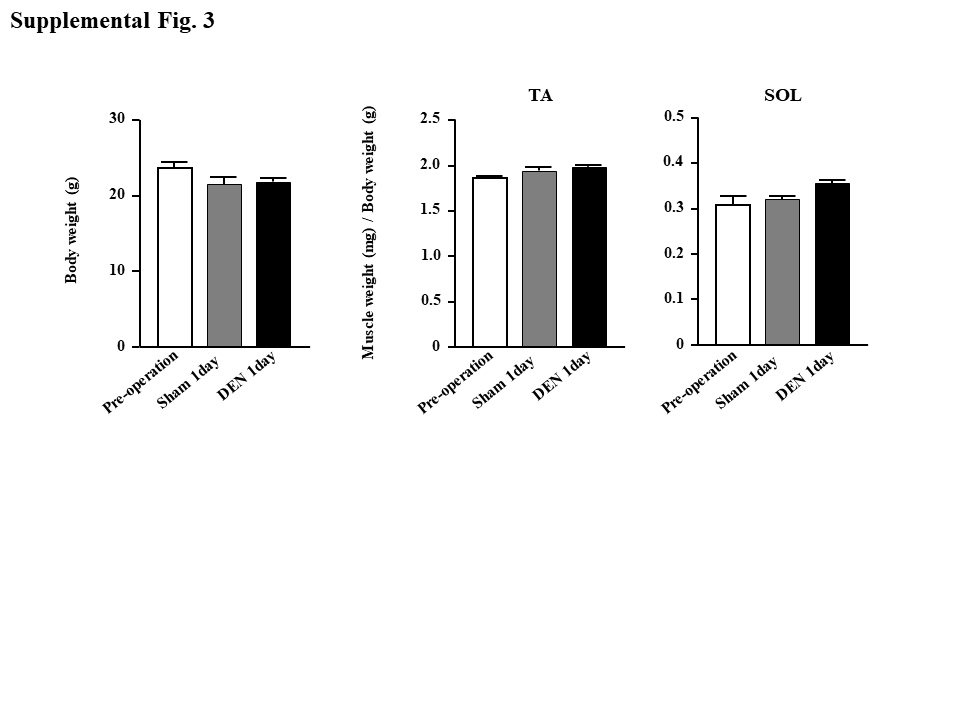

Supplement: S3 Fig — The body weight and TA and SOL muscles weight of the CON and DEN groups on pre- or post-operative day 1. (TIF) [file pone.0289185.s003.tif]

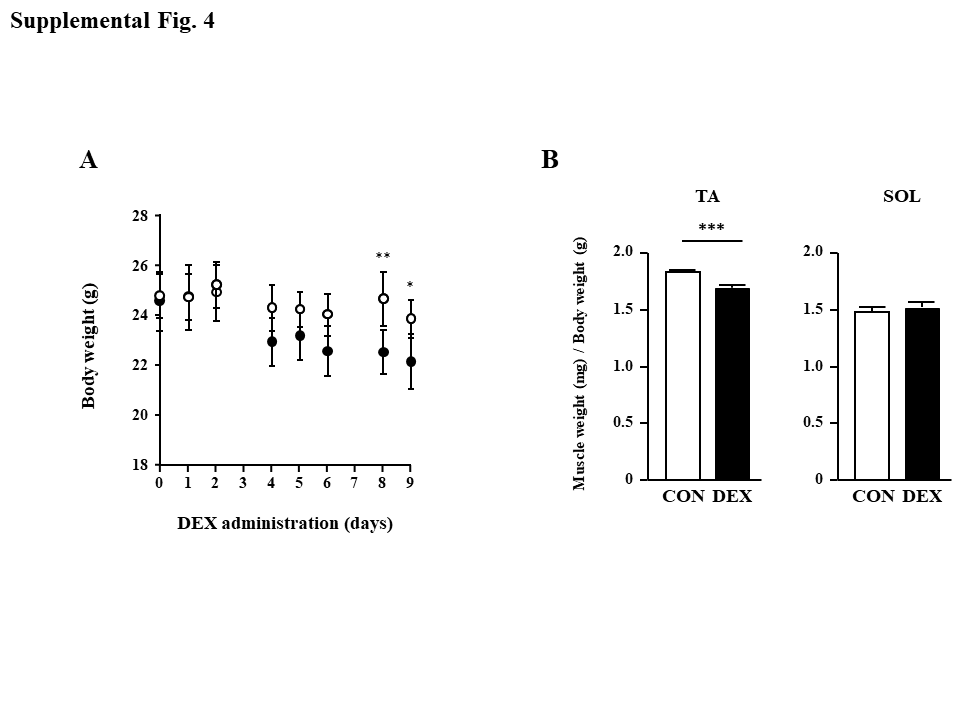

Supplement: S4 Fig — (A) Time course changes in the body weight. Open circles: CON group. Closed circles: DEN group. Data are presented as means ± standard error of the mean (SEM). *p < 0.05 and p < 0.01 (compared to the 0 day) by one-way repeated measures analysis of variance (ANOVA). (B) The weight in the TA and SOL muscles of the CON and DEX groups on administration day 10. (TIF) [file pone.0289185.s004.tif]

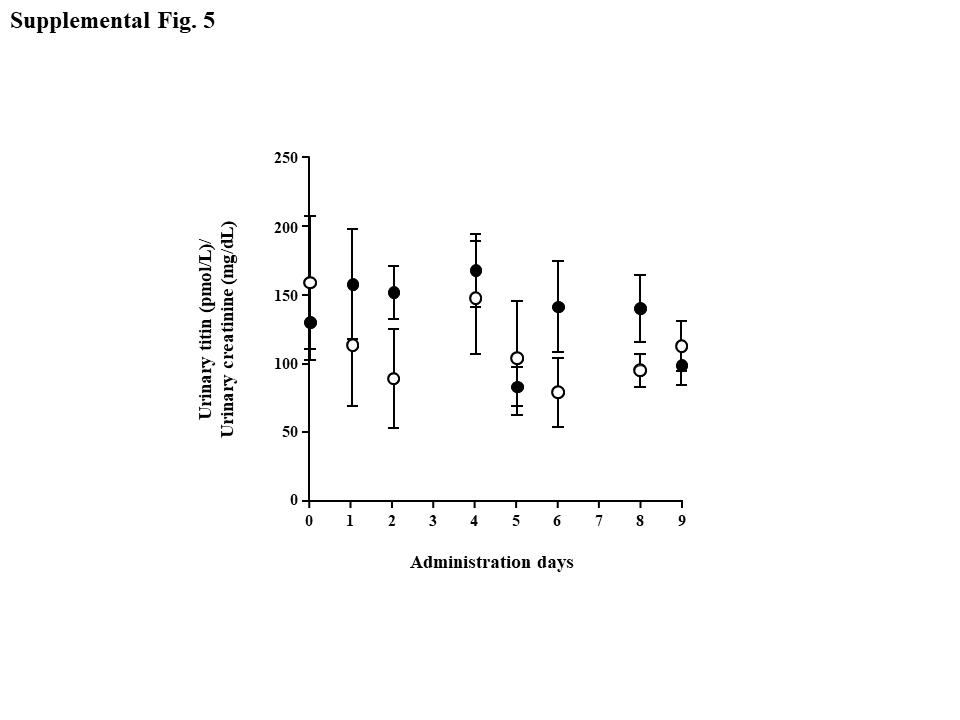

Supplement: S5 Fig — Time course changes in urinary N-titin/Cr levels. Open circles: CON group. Closed circles: DEX group. Data are presented as means ± standard error of the mean (SEM). (TIF) [file pone.0289185.s005.tif]

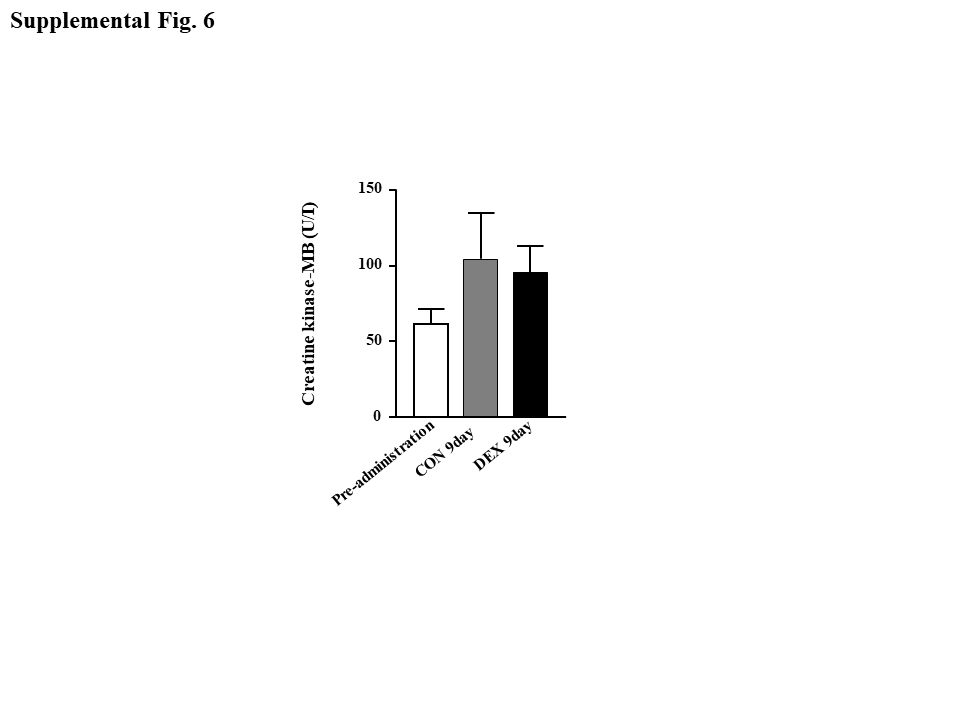

Supplement: S6 Fig — Serum CK-MB levels (U/L) in pre-administration, CON group and DEX group on day 9. (TIF) [file pone.0289185.s006.tif]
