# Supplementary material for: Urinary titin is not an early biomarker of skeletal muscle atrophy induced by muscle denervation in mice
Source: PLoS One. 2023 Aug 15;18(8):e0289185. doi: 10.1371/journal.pone.0289185 (PMC10426992; doi:10.1371/journal.pone.0289185)
Supplement: S2 File — (PDF) [file pone.0289185.s008.pdf]

Fig. 1C_raw_images

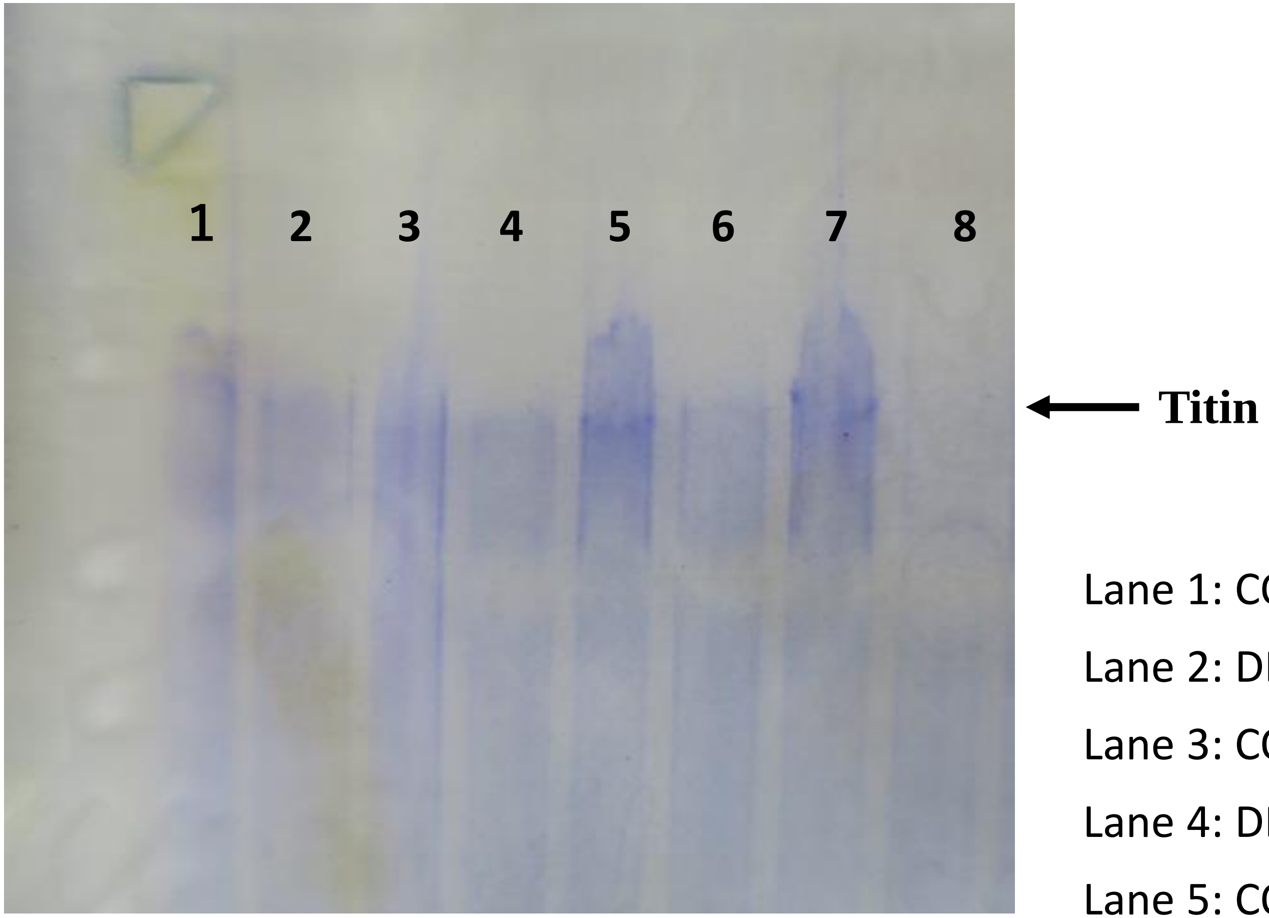

Lane 1: CON group SOL muscle

Lane 2: DEN group SOL muscle

Lane 3: CON group SOL muscle

Lane 4: DEN group SOL muscle

Lane 5: CON group SOL muscle

Lane 6: DEN group SOL muscle

Lane 7: CON group SOL muscle

Lane 8: DEN group SOL muscle
