# Supplementary material for: Urinary titin is not an early biomarker of skeletal muscle atrophy induced by muscle denervation in mice
Source: PLoS One. 2023 Aug 15;18(8):e0289185. doi: 10.1371/journal.pone.0289185 (PMC10426992; doi:10.1371/journal.pone.0289185)
Supplement: Attachment — Submitted filename: renamed_6b411.docx [file pone.0289185.s010.docx]

**Point-by-point responses to the Editor’s comments and the reviewers’ comments**

*Reviewer #1: In this manuscript, Tanihata and Minamisawa tested if urinary N-titin could be a potential early biomarker of skeletal muscle atrophy induced by sciatic nerve denervation in adult mice. They found that urinary levels of cleaved N-titin fragments were unaltered under the atrophic conditions, although full-length titin protein levels were reduced in muscle tissues. They found that the sarcomeric membrane was not injured by the denervation, probably through the upregulation of connexin 43. They concluded that cleaved N-titin is unlikely to be released into extracellular spaces from muscle cells and unaltered in the urine in the denervation model, and thus urinary cleaved N-titin is an unsuitable early biomarker for muscle atrophy.*

**We thank Reviewer 1 for his/her thorough review of the manuscript.　We have responded to each of Reviewer 1’s criticisms and have modified the manuscript accordingly. It is our hope that the revised version is now deemed acceptable.**

*-Figure 1: Since gene and protein levels were normalized by the total amounts of mRNAs and proteins, respectively, the titin levels should be relatively decreased in the atrophic muscle tissues. The authors explained that decreased titin protein was due to protein degradation mediated by E3-ubiquitin ligases such as MuRF1. However, there is a possibility that reduced mRNA levels also contributed to the reduction of protein levels in the atrophic muscle. How do they exclude this possibility?*

**We also had examined the points raised by reviewer1.**

**Titin protein levels decreased by denervation (Fig. 1C and D), but mRNA levels did not change (Fig. 1B).**

**Thus, the reduction of titin protein by denervation in this experiment appears to be due to proteolysis rather than a decrease in mRNA.**

*-Figure 3: the authors showed that urinary N-titin/Cr levels were markedly increased in both CON and DEN groups on postoperative day 1 compared to the non-operation groups. This could be due to the muscle damage by surgical incision. However, such upregulation is not observed in Supplemental Figure 2. The authors should explain this discrepancy.
Did the authors measure the serum creatine kinase levels? If it is unchanged in the denervation condition, they can conclude that muscle injury is not evident.*

**We believe that the reason for the lack of an increase in titin concentration in the initiaaly submitted version of Supplemental Figure 2 could be maily due to the different sensitivity of the kits used. In the additional experiment, serum CK-MB levels were transiently elevated immediately after surgery. Therefore, these results suggest that the increase in urinary titin concentration is due to muscle damage in the early stages of muscle atrophy.**

*-Supplemental Figures 1 & 2: supplemental figures should be included into the main figures so that the readers can easily compare the sensitivities of ELISA kits for the detection of titin protein.*

**Following the suggestion of reviewer 1, supplemental figures 1 and 2 were newly included in the figure 3.**

*Reviewer #2: The manuscript describes that the N-terminal titin fragment was not increased in the urine despite reduction of the titin protein levels in atrophic muscles after sciatic nerve transection in mice. The authors concluded that the urinary titin fragment is not a suitable biomarker for detecting the early stage of skeletal muscle atrophy.*

**We thank Reviewer 2 for his/her thorough review of the manuscript. We have responded to each of Reviewer 2’s criticisms and have modified the manuscript accordingly. It is our hope that the revised version is now deemed acceptable.**

**Following the suggestion of reviewer 2, we also examined urinary titin levels during muscle atrophy induced by dexamethasone administration.**

*There are several concerns as below:*

*1) As authors describe, skeletal muscle atrophy is a phenomenon resulted from various causes including disuse, denervation, aging, cachexia, as well as muscle diseases such as muscular dystrophies. Therefore, the underlying mechanism is supposed to differ depending on its cause. It should be clearly stated that sciatic nerve transection is a model of denervation and that this study was conducted to examine the efficacy of the urinary N-terminal titin as a biomarker detecting denervation-induced atrophy. The term skeletal muscle atrophy is used loosely throughout and the discussion on interpretation of the results seems imprecise. For instance, they discussed comparison with nonsurgical, critically ill patients, but it seems that the underlying pathology is not denervation.*

**In according with the remarks of Reviewer 2, we described that the muscle atrophy used this study was due to denervation.**

*2) Does cleaved N-terminal titin occur in skeletal muscle? In Fig.1C western blot analysis did not clearly show the N-terminal titin fragment. Could titin's cleavage occur outside the skeletal muscle after leakage?***This question of reviewer 2 is very important and is what we would like to know. One possible explanation for the lack of N-titin fragments detected in urine is that either muscle atrophy does not produce N-titin fragments in the first place, or that N-titin fragments are produced in muscle atrophy but remain intracellular and do not appear in urine. To detect whether N-titin fragments are produced in atrophied skeletal muscle cells or not, we extracted proteins from TA and SOL using SYPRO Ruby and examined for the presence of N-titin fragments (Reference Figure 1 below). As a result, no clear band was observed around 21.4 kD, the molecular size of the N-titin fragment. Although this detection method clearly indicates that the amount of N-titin fragments is low, we believe that it is not yet possible to conclude that cleavage is not actually occurring. To clarify whether titin is cleaved by muscle atrophy, a new antibody that detects the N-titin fragment needs to be generated. This will take time, and we hope to clarify this in future studies.**

*3) The authors showed upregulation of connexin 43 in denervated muscle. They speculate the N-titin fragment cannot go through the connexin hemichannels because of its molecular size. If it is the case, why EBD uptake was not observed?*

**Although connexin 43 expression is elevated in denervated skeletal muscle, the elevated level is only about 1.5-fold, which is clearly lower than the level of connexin 43 expression in the heart. Thus, although connexin 43 expression levels are elevated in atrophied skeletal muscle, it is unlikely to play a significant role in EBD uptake. Moreover, if EBD can pass through connexin 43, then EBD should also be taken up in the normal heart, but no such images were observed in normal heart.**

*To address the above-mentioned questions, it is necessary to analyze the status of titin in the circulating blood under skeletal muscle atrophy caused by different etiologies.*

**According to the Reviewer 2’s remark, as an additional experiment, we examined urinary titin levels in a dexamethasone-induced muscle atrophy model in Supplemental Figures 4 to 6. The results showed that dexamethasone caused muscle atrophy but did not alter the level of urinary titin.**

*Reviewer #3: The study by Tanihata and Minamisawa examined the importance of titin in skeletal muscle atrophy during denervation. The results show that there is no increase in the expression of urinary/muscle titin during denervation. Therefore, the authors highlight that the titin levels was not reflected lower limb muscle atrophy. Unfortunately, most part of this work relies on muscle atrophy following denervation, an early atrophy model.*

**We thank Reviewer 3 for his/her thorough review of the manuscript.　We have responded to each of Reviewer 3’s criticisms and have modified the manuscript accordingly. It is our hope that the revised version is now deemed acceptable.**

**Following the suggestion of reviewer 3, we also examined urinary titin levels during muscle atrophy induced by dexamethasone administration.**

*Me specific concerns are outlined below.*

*The authors should examine other early muscle atrophy models, such as disuse, and disease (or dexamethasone), because this manuscript focusing early muscle atrophy.*

**Reviewer 2 made similar comments, so we examined urinary titin levels in a dexamethasone-induced muscle atrophy model. As shown in Supplemental Figures 4 to 6, the results showed that dexamethasone caused muscle atrophy but did not alter the level of urinary titin.**

*Since the greatest increase in the titin was observed 1 day after denervation, inflammation should be considered. What was the degree of muscle atrophy following 1 day?*

**We measured muscle weights on the first day after denervation. The results showed no change in muscle weight compared to the pre-operative or CON groups. These results suggest that the increase in urinary titin levels one day after denervation was not due to muscle atrophy.**

*How was titin levels in other hindlimb muscle?*

**We have observed that titin in the gastrocnemius muscle is also reduced by denervation. This data includes Supplemental Figures 1**

*Why the author did not measure connexin 43 in the SOL muscle?*

**We also measured the levels of connexin43 in SOL muscle. The results showed a similar trend to TA muscles, but the bands were too fine due to the low amount of loading protein, so we did not include it in the main figure or supplemental figure.**

**Therefore, this figure is shown below for the reviewer only (Reference Fig. 2 as below).**

*Minor
The authors should describe "expression" only for genes, not proteins.*

**According to Reviewer 2’s suggestion, we removed the “expression” for proteins.**

*- Figures: Show marker position for each blot image.*

**The molecular weights were added in the main Fig 4.**

**Molecular weights ware also added in the PDF files of original blot and gel images.**
